# Remifentanil patient controlled analgesia versus epidural analgesia in labour. A multicentre randomized controlled trial

**DOI:** 10.1186/1471-2393-12-63

**Published:** 2012-07-02

**Authors:** Liv M Freeman, Kitty WM Bloemenkamp, Maureen TM Franssen, Dimitri NM Papatsonis, Petra J Hajenius, Marloes E van Huizen, Henk A Bremer, Eline SA van den Akker, Mallory D Woiski, Martina M Porath, Erik van Beek, Nico Schuitemaker, Paulien CM van der Salm, Bianca F Fong, Celine Radder, Caroline J Bax, Marko Sikkema, M Elske van den Akker-van Marle, Jan MM van Lith, Enrico Lopriore, Renske J Uildriks, Michel MRF Struys, Ben Willem J Mol, Albert Dahan, Johanna M Middeldorp

**Affiliations:** 1 Department of Obstetrics and Gynaecology, Leiden University Medical Centre, Leiden, the Netherlands; 2 Department of Obstetrics and Gynaecology, University Medical Centre Groningen, Groningen, the Netherlands; 3 Department of Obstetrics and Gynaecology, Amphia hospital, Breda, the Netherlands; 4 Department of Obstetrics and Gynaecology, Academic Medical Centre, Amsterdam, the Netherlands; 5 Department of Obstetrics and Gynaecology, HagaZiekenhuis, Den Haag, the Netherlands; 6 Department of Obstetrics and Gynaecology, Reinier de Graaf Gasthuis, Delft, the Netherlands; 7 Department of Obstetrics and Gynaecology, Onze Lieve Vrouwe Gasthuis, Amsterdam, the Netherlands; 8 Department of Obstetrics and Gynaecology, Radboud University Nijmegen Medical Centre, Nijmegen, the Netherlands; 9 Department of Obstetrics and Gynaecology, Maxima Medical Centre, Veldhoven, the Netherlands; 10 Department of Obstetrics and Gynaecology, St. Antonius hospital, Nieuwegein, the Netherlands; 11 Department of Obstetrics and Gynaecology, Diakonessenhuis, Utrecht, the Netherlands; 12 Department of Obstetrics and Gynaecology, Meander Medical Centre, Amersfoort, the Netherlands; 13 Department of Obstetrics and Gynaecology, Zaans Medical Centre, Zaandam, the Netherlands; 14 Department of Obstetrics and Gynaecology, St Lucas Andreas hospital, Amsterdam, the Netherlands; 15 Department of Obstetrics and Gynaecology, VU Medical Centre, Amsterdam, the Netherlands; 16 Department of Obstetrics and Gynaecology, ZiekenhuisGroepTwente, Almelo, the Netherlands; 17 Department of Medical Decision Making, Leiden University Medical Centre, Leiden, the Netherlands; 18 Department of Pediatrics, Leiden University Medical Centre, Leiden, the Netherlands; 19 Department of Anesthesiology, University Medical Centre Groningen, Groningen, the Netherlands; 20 Department of Anesthesiology, Leiden University Medical Centre, Leiden, the Netherlands

**Keywords:** Analgesia, Labour, Remifentanil, Patient controlled analgesia, Epidural

## Abstract

**Background:**

Pain relief during labour is a topic of major interest in the Netherlands. Epidural analgesia is considered to be the most effective method of pain relief and recommended as first choice. However its uptake by pregnant women is limited compared to other western countries, partly as a result of non-availability due to logistic problems. Remifentanil, a synthetic opioid, is very suitable for patient controlled analgesia. Recent studies show that epidural analgesia is superior to remifentanil patient controlled analgesia in terms of pain intensity score; however there was no difference in satisfaction with pain relief between both treatments.

**Methods/design:**

The proposed study is a multicentre randomized controlled study that assesses the cost-effectiveness of remifentanil patient controlled analgesia compared to epidural analgesia. We hypothesize that remifentanil patient controlled analgesia is as effective in improving pain appreciation scores as epidural analgesia, with lower costs and easier achievement of 24 hours availability of pain relief for women in labour and efficient pain relief for those with a contraindication for epidural analgesia.

Eligible women will be informed about the study and randomized before active labour has started. Women will be randomly allocated to a strategy based on epidural analgesia or on remifentanil patient controlled analgesia when they request pain relief during labour. Primary outcome is the pain appreciation score, i.e. satisfaction with pain relief.

Secondary outcome parameters are costs, patient satisfaction, pain scores (pain-intensity), mode of delivery and maternal and neonatal side effects.

The economic analysis will be performed from a short-term healthcare perspective. For both strategies the cost of perinatal care for mother and child, starting at the onset of labour and ending ten days after delivery, will be registered and compared.

**Discussion:**

This study, considering cost effectiveness of remifentanil as first choice analgesia versus epidural analgesia, could strongly improve the care for 180.000 women, giving birth in the Netherlands yearly by giving them access to pain relief during labour, 24 hours a day.

**Trial registration number:**

Dutch Trial Register NTR2551, http://www.trialregister.nl

## Background

Epidural analgesia is considered to be the most effective method of pain relief during labour and is recommended as first method of pain relief by the Dutch Societies of Gynecologists and Anesthetists [1,2]. In the Netherlands its uptake by pregnant women in labour of all ethnicities is still limited (11.3% in 2008 but in the last years increasing with 1-2% per year), compared with other western countries, partly as a result of non-availability due to logistic problems. This is an undesirable situation, especially since the number of women asking for pain relief during labour is increasing. There is also need for a safe alternative for women who cannot receive epidural analgesia because of contraindication for epidural analgesia.

The availability and uptake of epidural analgesia during labour varies significantly between countries, for example approximately 20% of women in the UK and 58% of women in the USA use this form of pain relief. There is considerable variation in the availability of epidural analgesia within the UK as in the Netherlands [3].

There are situations in which epidural analgesia is contra-indicated. In these cases intramuscular or intravenous opioids provide an alternative. Variation is also present in the use of opioids during labour, reported numbers range from 5-66%. In the last update of the Cochrane review “Parenteral opioids for maternal pain management in labour” the authors recommend a pragmatic large randomized controlled trial to compare pain relief using an opioid to other methods of pain relief to collect data on maternal satisfaction, co-interventions and maternal and neonatal outcome prospectively [4].

At present in the Netherlands, pain relief during labour is of major interest and an important topic for pregnant women, health care providers and politicians, as is pointed out in the publication of the Steering Committee Pregnancy and Birth installed by the Dutch Ministry of Health, Welfare and Sports [3]. One of the advices is that all Dutch women in labour should have access to adequate pain relief. The working party of the Dutch guideline "Pain relief during labour" recommends using remifentanil patient controlled analgesia (PCA) only in controlled setting and recommends a large trial. Nevertheless, over one third of Dutch hospitals use remifentanil PCA on labour wards. Possible explanations are that the presence of an anesthetist for this type of analgesia is not required and that administration of remifentanil is quicker and less invasive than epidural analgesia. Literature study reveals that this does not only apply to the Dutch obstetrical system, which differs from other western countries because of a higher percentage of women under the care of community midwives and of home-births.

The most commonly used opioid is intramuscular pethidine. However, its analgesic effectiveness is widely challenged [5-7]. Remifentanil is a synthetic opioid (anilidopiperidine) with direct agonist action specifically on μ-opioid receptors [8]. The rapid onset and offset of the drug make remifentanil very suitable for administration via patient controlled analgesia (PCA), which can be used for analgesia during labour. Placental transfer of remifentanil does occur but appears to be rapidly metabolized, redistributed, or both. There were no adverse neonatal or maternal effects, only mild maternal sedation and respiratory changes [9]. There have been multiple clinical studies on the use of remifentanil in women in labour [10-22].

Two studies address pain relief scores of remifentanil PCA (patient controlled analgesia) compared to epidural analgesia, although both had limitations. Volmanen et al. limited the observation period to only one hour. Douma et al. recorded pain relief scores as a secondary outcome measure in a study powered to investigate difference in pain scores. Both studies showed that in terms of pain scores (pain-intensity), epidural analgesia is superior tot remifentanil PCA. However, there was no difference in the pain appreciation scores between both treatments [23,24].

## Methods/Design

### Aims

The objective of this study is to test the hypothesis that remifentanil PCA is as effective as epidural analgesia with respect to patient satisfaction and pain appreciation scores, with lower costs and possibly the benefit of easier achievement of 24 hours availability of pain relief for women in labour.

### Participants/eligibility criteria

All pregnant women in the participating hospitals will be informed of the trial at antenatal visits in the third trimester. They can participate in the trial if they are healthy or have a mild systemic disease (ASA physical status 1 or 2) and are 18 years or older with a gestational age >32 weeks. Randomization takes place before active labour has started, at antenatal visits in the third trimester or at admission on the ward before induction. Exclusion criteria are hypersensitivity for any of the products used or if there is a contraindication for epidural analgesia.

### Procedures, recruitment, randomization, collection of baseline data

The study will be a multicentre randomized controlled study. The study will be performed within the Dutch Consortium for Studies in Women’s Health and Reproductivity. Participating hospitals can be district, teaching or third referral hospitals. Before entry into the study, women are informed about the aims, methods, reasonably anticipated benefits and potential hazards of the study. They are informed that their participation is voluntary and that they may withdraw consent to participate at any time during the study. Choosing not to participate will not affect care. In every centre an independent gynaecologist will be available for more detailed information both for patients and colleagues if required.

After giving sufficient information, written informed consent is obtained. The consent form must be signed before performance of any study-related activity. After obtaining informed consent women will be randomized and will be informed on the assigned method of pain relief before labour starts (as in usual care). They are only given pain relief during labour at their request or if a medical reason should arise.

Randomization will be stratified for centre and parity. We will apply block randomization with a fixed block size. Randomization will be performed through a web-based database located in the central data collection unit in the AMC in Amsterdam. Women will be randomly allocated to receive remifentanil PCA or epidural analgesia when they request pain relief during labour. There will be no blinding, as this is not possible due to the nature of the two treatment methods.

Baseline demographic, past obstetric and medical histories, including ASA physical status, will be recorded for all women.

All details of delivery and health care received in the ten days after delivery are recorded in the case record form that is accessible through a website http://www.studies-obsgyn.nl/ravel.

### Interventions

After giving informed consent women will be randomized to receive remifentanil PCA or epidural analgesia during labour if they request pain relief during labour. Parturients randomized to intravenous remifentanil will receive a 30 μg loading dose and boluses of 30 μg with a 3 minute lockout time. We decided on a flexible bolus dose. In case of insufficient pain relief the bolus can be increased to 40 μg or decreased to 20 μg in case of excessive side effects. Parturients randomized to epidural analgesia will receive epidural analgesia according to local protocol.

### Outcome measures

The main outcome parameter is pain appreciation, i.e. satisfaction with pain relief. Women will be asked to express their level of satisfaction with pain relief every 15 min during the first hour and hourly after that. This will be scored on a visual analogue scale (VAS) ranging from 1 (highly dissatisfied) to 10 (highly satisfied).

Secondary endpoints are pain scores, scored on a visual analogue scale ranging from 1 (no pain) to 10 (worst imaginable pain), maternal side effects, mode of delivery and maternal and neonatal mortality and morbidity.

### Economic evaluation

#### General consideration

The results of the study will provide insight on whether remifentanil PCA in women in labour will reduce costs as compared to epidural analgesia, assuming there will be equivalence in pain appreciation of both methods. At present, no clinical study has been published or undertaken to investigate this issue. An estimation of costs for remifentanil PCA versus epidural analgesia shows a decrease of 64 euro per patient. The difference in costs is due to the extra costs of anesthetic staff and nurses, required when epidural analgesia is given.

#### Cost analysis

Economic analysis

The economic analysis will be performed from a short-term healthcare perspective. Anticipating on equality in pain appreciation scores the economic analysis will be a cost minimization analysis. For both strategies the cost of perinatal care for mother and child, starting at the onset of labour and ending ten days after delivery, will be registered and compared (without discounting). The costs consist of costs of delivery/childbirth (course and mode of delivery), postnatal maternal care (hospitalization, outpatient visits), neonatal care (admission to NICU/neonatology ward, outpatient visits) and primary care (midwife, general practitioner, maternity care).

Volumes of hospital care are measured prospectively alongside the clinical study in all participating centres as part of the case record form. Health resource use outside the hospital will be recorded by questionnaires filled out by the patients. Costs of delivery/childbirth will be based on cost price analysis. Other resource use (hospital days, outpatient visits and primary care) will be valued using standard prices [25].

### Follow up of women and infants

Details of admission of women and newborns will be recorded as will maternal and neonatal complications. Long term follow up is at present not part of this study.

### Statistical issues

#### Sample size

The sample size is calculated based on the primary outcome measure pain appreciation. We hypothesize that there is no difference in pain appreciation with the two sided test (alfa = 0.05, power (1-beta = 0.9). In this non-inferiority design in each group 102 women have to be treated to exclude a potential clinical relevant difference of 10% (10 point scale, estimated SD 2.2). Allowing for 10% and 30% cross-over/ non-compliance in the control group and experimental group respectively, 568 patients are required. We estimate that in the group of pregnant women who are willing to participate in the study 50% will actually need pain relief. This in contrast to the whole Dutch pregnant population, which is known for a low uptake of pain relief during labour. Therefore 1136 women have to be randomized. In case of missing data on the primary endpoint, we will extend the number of women to be recruited accordingly.

#### Data analysis

Data will be analyzed according the intention to treat principle. First, the remifentanil and epidural group will be compared. Relative risks and 95% confidence intervals will be calculated for the relevant outcome measures. Categorical variables will be tested with the Chi-square test or Fisher’s exact test. Continuous variables will be tested with the Mann–Whitney *U* test. Time to delivery will be assessed using Kaplan-Meier analysis. In case of equivalence between outcomes, the analysis will be repeated on a par protocol basis. Subsequently, planned subgroup analysis will be done for nulliparous versus parous women, previous caesarean section, preterm labour (32–34 weeks and 34–37 weeks) and term labour (37–42 weeks), spontaneous versus induced labour, maternal educational level, maternal age (under 35 years versus over 35 years) and multiple pregnancy. We will then use decision analysis to evaluate which intervention strategy, i.e. remifentanil or epidural analgesia is preferred in women who need analgesia during labour.

#### Interim analysis

No interim analysis will be performed. Because of the suggested design of the trial where equivalence is expected all 1136 women have to be randomized in order to achieve sufficient power. Both remifentanil PCA and epidural analgesia are widely used in the Netherlands as pain relief during labour and no adverse events have been recorded to date.

Serious Adverse Events (SAEs) and Suspected Unexpected Serious Adverse Reactions (SUSARs) will be reported to a Data Safety Monitoring Committee (DSMC). The DSMC can order to perform an interim analysis and, if indicated, terminate the trial prematurely.

#### Ethical considerations

This study is approved by the National Central Committee on Research involving Human Subjects (CCMO - NL34262.058.10.), by the ethics committee of the Leiden University Medical Centre (Ref. No. P10-240) and by the boards of management and ethics committees of all participating hospitals.

## Discussion

In the Netherlands uptake of epidural analgesia is lower than in surrounding western European countries. This can be partly due to our obstetrical system; a large number of women under the care of community midwives and about 25% of all births take place at home. Epidural analgesia is recommended as first method of pain relief by the Dutch Societies of Gynecologists and Anesthetists. In daily practice not every labour ward in the Netherlands has 24 hour availability of epidural analgesia. One of the alternatives is remifentanil PCA. Over one third of all hospitals use remifentanil as pain relief during labour. With this study we aim to test the hypothesis that remifentanil PCA and epidural analgesia are equivalent in pain appreciation with possible fewer costs. The outcome of this study could improve the care for over 180.000 women giving birth in the Netherlands yearly.

## Abbreviations

ASA, American Society of Anesthesiologists; PCA, Patient controlled analgesia; DSMB, Data safety monitoring board.

## Competing interests

The authors declare that they have no competing interests.

## Authors’ contributions

AM, KB, AD, MS, JvL, and BWM were involved in the conception and design of the study. LF, AM, KB, AD, MS, JvL, and BWM drafted the manuscript. All authors mentioned in the manuscript are members of the RAVEL-trial study group. They are local investigators at the participating centers. All authors read, edited and approved the final manuscript.
